# Postural Dynamics Are Associated With Cognitive Decline in Parkinson's Disease

**DOI:** 10.3389/fneur.2018.01044

**Published:** 2018-12-05

**Authors:** Annette Pantall, Piriya Suresparan, Leanne Kapa, Rosie Morris, Alison Yarnall, Silvia Del Din, Lynn Rochester

**Affiliations:** ^1^Clinical Ageing Research Unit, Institute of Neuroscience, Newcastle University Institute of Ageing, Newcastle upon Tyne, United Kingdom; ^2^Department of Neurology, Oregon Health and Science University, Portland, OR, United States; ^3^The Newcastle upon Tyne NHS Foundation Trust, Newcastle upon Tyne, United Kingdom

**Keywords:** posture, Parkinson's, cognition, balance, depression, longitudinal, accelerometer

## Abstract

Early features of Parkinson's disease (PD) include both motor and cognitive changes, suggesting shared common pathways. A common motor dysfunction is postural instability, a known predictor of falls, which have a major impact on quality of life. Understanding mechanisms of postural dynamics in PD and specifically how they relate to cognitive changes is essential for developing effective interventions. The aims of this study were to examine the changes that occur in postural metrics over time and explore the relationship between postural and cognitive dysfunction. The study group consisted of 35 people (66 ± 8years, 12 female, UPDRS III: 22.5 ± 9.6) diagnosed with PD who were recruited as part of the Incidence of Cognitive Impairment in Cohorts with Longitudinal Evaluation—PD Gait (ICICLE-GAIT) study. Postural and cognitive assessments were performed at 18, 36, and 54 months after enrolment. Participants stood still for 120 s, eyes open and arms by their side. Postural dynamics were measured using metrics derived from a single tri-axial accelerometer (Axivity AX3, York, UK) on the lower back. Accelerometry metrics included jerk (derivative of acceleration), root mean square, frequency, and ellipsis (acceleration area). Cognition was evaluated by neuropsychological tests including the Montreal Cognitive Assessment (MoCA) and digit span. There was a significant decrease in accelerometry parameters, greater in the anteroposterior direction, and a decline in cognitive function over time. Accelerometry metrics were positively correlated with lower cognitive function and increased geriatric depression score and negatively associated with a qualitative measure of balance confidence. In conclusion, people with PD showed reduced postural dynamics that may represent a postural safety strategy. Associations with cognitive function and depression, both symptoms that may pre-empt motor symptoms, suggest shared neural pathways. Further studies, involving neuroimaging, may determine how these postural parameters relate to underlying neural and clinical correlates.

## Introduction

Parkinson's disease (PD) is a common progressive neurodegenerative disease with a UK incidence of 84 per 100,000 in adults over 50 years (1). Clinical characterizations include both motor and non-motor manifestations, indicative of a multisystem neurodegenerative disease (2). Common motor symptoms include resting tremor, bradykinesia, rigidity, and postural instability (3). Postural instability is clinically important as it is a predictor of falls, which impact on quality of life (4). Falls may result in injury, leading to possible loss of functional independence, institutionalization and a poor quality of life (5, 6). Previous studies indicate that 38–68% of people with PD are subject to falls, 25% of which have two or more falls every 6 months (7–10).

Postural instability is classically defined as the inability to maintain the center of mass within its base of support. Clinically, the retropulsion (pull) test is applied to assess postural instability (11). Postural sway is an indicator of postural instability and a measure of the sensorimotor control loop that regulates standing balance (12). Postural sway is greater in fallers than non-fallers, therefore is an important clinical marker (13). The traditional method of recording postural sway involves tracking the center of pressure using a force platform (14). More recently, body-worn sensors (e.g., accelerometers, gyroscopes, magnetometers, insole pressure sensors) have been developed which permit measurements to be made outside a laboratory setting (12). Strong correlations have been found between accelerometer parameters (postural dynamics) and force-platform derived center of pressure data, thereby validating the accelerometer as a method for assessing postural sway (15, 16). Accelerometry metrics during the first 30 s of standing have been reported to be discriminative of PD (17).

Mild cognitive impairment (MCI) is a non-motor feature present in over 20% of patients at initial diagnosis (18). Cognitive function deteriorates with disease progression, with a >2-fold increase in MCI reported over 3 years (19). Cognitive decline in PD is associated with dysfunction of both dopaminergic and cholinergic pathways as well as increased Lewy bodies and possible vascular pathology (20, 21). Performing a cognitive task while standing has been reported to increase postural parameters and by implication, postural instability in people with PD (22–24) compared to healthy older adults. This suggests a “posture second” prioritization, associated with decreased attentional resources available. With cognitive decline, one might anticipate increased postural instability and correlation between cognitive function and postural parameters. Studies have reported a relationship between cognitive changes and postural instability (25, 26) in addition to an association with fall risk (27, 28). This may reflect common shared pathways or adverse events affecting multiple networks. Another common non-motor symptom in PD is depression which has been observed in, on average, 40% of people with PD (29). In PD, Lewy bodies have been found in many subcortical nuclei including the locus coeruleus (30). The locus coeruleus is associated with arousal and also muscle tone critical for postural stability. Patients with depression have been reported to have greater changes in the locus coeruleus compared to non-depressed patients (31). Several studies have reported an association between depression and postural instability (32, 33). Changes in the locus coeruleus and noradrenergic system may partly account for the association between posture and depression. In PD, motor and non-motor features do not exist as separate entities, but rather display interactions, which warrant further investigation.

Information regarding the time-course of postural sway in people with PD is limited as few longitudinal postural studies have been undertaken. Understanding the progression of postural sway may improve our understanding of underlying mechanisms. Exploring the relationship between postural dynamics and cognitive function will inform us of the interaction between the motor and non-motor systems and the effect of diminishing attentional resources on postural stability. Examining the association between depression and postural instability may illuminate the effect of shared pathways on these motor and non-motor features. Clinically, understanding mechanisms underlying postural instability is important given the impact postural control has on falls, gait, and mobility. The main aim of this longitudinal study was to explore how postural dynamics change during quiet standing in people with PD over 36 months. Postural dynamics were determined from accelerometer recordings over the course of a 120 s standing balance test. The hypothesis was that measures of postural dynamics would increase over the 36 months period, indicating increased postural instability. A further hypothesis was that the greatest change in parameters would occur during the first 30 s of the postural task. The second aim was to investigate the relationship between postural instability and global cognition and depression. The hypothesis was that there would be a significant relationship between motor and non-motor features.

## Methods

### Participants and Clinical Assessments

The study group consisted of 35 people recruited from the Incidence of Cognitive Impairment in Cohorts with Longitudinal Evaluation—Parkinson's disease Gait study (ICICLE-PD GAIT) study (34). The participants underwent a baseline assessment, followed by cognitive and postural assessments at 18, 36, and 54 months. Not all participants underwent a baseline postural assessment; therefore, this study does not include baseline measurements. Participants were assessed at the Clinical Aging Research Unit, Newcastle University. The study was approved by the Newcastle and North Tyneside research ethics committee and conducted according to the declaration of Helsinki. All participants signed an informed consent form prior to testing.

The exclusion criteria included any neurological (other than PD), orthopedic or cardiothoracic condition that may adversely have affected the participant's gait or safety. Additional exclusion factors included cognitive impairment (Mini Mental State Exam (MMSE) ≤ 24) and difficulties comprehending English. Parkinson's disease was diagnosed according to the UK Parkinson's Brain Bank criteria (35).

At each assessment, demographic, clinical, and cognitive information were collected. The Hoehn and Yahr scale was used to measure the motor symptom severity in PD participants (36). The Movement Disorder Society Unified Parkinson's Disease Rating Scale (MDS—UPDRS) Part III (37) assessed motor function in PD (0-no motor symptoms, 132-severe motor symptoms). Balance confidence was assessed using the Activities Balance Self Confidence Scale (ABC), with a score of 0 indicating no confidence and a score of 100 indicating complete confidence in balance when performing various activities (38). Cognitive tests included the Montreal Cognitive Assessment (MoCA) (39) for global cognition (score range 0–30) with a score of 26–30 indicating normal cognitive function and < 26 suggesting cognitive impairment. The Wechsler Forward Digit Span tested working memory (40), the average number of digits a healthy adult can recall being 7 ± 2 (41). The short Geriatric Depression Scale (GDS) (42) was used as a measure of depressive symptoms (score range 0–15). A score of 0–4 is considered normal, 5–8 indicates mild depression and a score of 12–15 indicates severe depression.

### Standing Balance Test

The standing balance test was carried out an hour after medication intake. Participants stood in an upright position with their feet positioned within a predefined area (400 mm wide × 600 mm long), with their hands by their side (43) and looking straight ahead for 120 s. There were no foot placement restrictions and the participants wore their shoes during the test. The recording began 3 s after the participant had understood the instructions of the tests.

### Equipment

A tri-axial accelerometer-based monitor (Axivity AX3, York, UK) on the lower back (L5) recorded acceleration at a sampling rate of 100 Hz (17). The accuracy of the accelerometer clock was ±20 parts per million, the resolution was 0.976 mg, the weight of the accelerometer was 9 g with dimensions of 6.0 × 21.5 × 31.5 mm. The Axivity AX3 accelerometer has been validated for recording high resolution movement (44). The accelerometer was attached to the skin with a hydrogel adhesive and Hypafix bandage.

### Data Processing

The data processing and analysis have previously been described by Del Din et al. (17). In summary, the data were downloaded to a computer and analyzed by customized MATLAB (R2015a, Mathworks, Natick, MA, USA.) algorithms. Analyses included rotation of the data into anteroposterior (AP), mediolateral (ML), and vertical accelerations using the Moe-Nilssen transformation (45). The following features were then extracted:

a) Jerk (m^2^.s^−5^): the rate of change of acceleration (46).
  Jerk was calculated for AP and ML and combined axes.
b) Root mean square [RMS (m.s^−2^)]: a measure of amplitude (46).
  RMS was calculated for AP and ML and combined axes.
c) Frequency (Hz): 95% of power of the acceleration power spectrum below frequency.
  Frequency was estimated for AP and ML axes (46).
d) Ellipsis (m^2^.s^−4^): the area comprising 95% of the AP and ML acceleration trajectories (14).

The four features were selected based on previous studies by Mancini et al. (16) who showed these to be sensitive to disease progression and disease discrimination. Additionally, these features can discriminate between different postural tasks in healthy older adults (47).

All accelerometer features were then determined for the following three phases of standing; the first 30 s, the first 60 s and the entire 120 s.

### Statistical Analysis

The data were analyzed using SPSS software (v21; IBM, Chicago, IL, USA). Outliers >2 standard deviations from the mean were removed from the datasets. Linear mixed-effects models were applied to determine the main effects of time-points (18, 36, 54 months), axes (AP, ML, combined) and phase (30, 60, 120 s) and their interaction effects on accelerometry parameters (*p* < 0.05). The RANDOM subcommand was used to model the covariance between the three axes and between the three phases. Sidak corrections for multiple comparisons were applied. The Friedmann test was applied to non-parametric Levodopa equivalent daily dose (LEDD), Hoehn and Yahr, MDS-UPDRS III, ABC, MoCA, digit span and GDS scores. The Wilcoxon signed-rank test compared different time-points. Spearman's rank correlation was used to examine cross-sectional associations between postural parameters and the ABC, MoCA, and GDS scores. Pearson's product-moment correlation coefficient determined associations between changes in postural parameters and cognitive parameters between time-points. The magnitude of effect of the correlation coefficients was defined by the following: *r* < 0.10: negligible; 0.10 ≤ *r* < 0.30: weak; 0.30 ≤ *r* < 0.50: moderate; *r* ≥ 0.50: strong (48).

## Results

### Demographic and Clinical Data

Table 1 lists demographic and clinical information at baseline, 18, 36, and 54 months. There was a greater number of males in the cohort. Although, all participants satisfied the inclusion criterion of MMSE>24, the MoCA at Baseline ranged from 20 to 30 with 10 participants having MoCA scores ≤ 24. There was a significant effect of time for LEDD, Hoehn, and Yahr stage and MDS-UPDRS III [X${}_{\left(3\right)}^{2}$ = 78.0, *p* < 0.001; X${}_{\left(3\right)}^{2}$ = 18.1, *p* < 0.001; X${}_{\left(3\right)}^{2}$ = 41.0, *p* < 0.001, respectively]. H&Y was significantly greater at 18 months compared to baseline (*Z* = −2.5, *p* = 0.012). LEDD increased significantly between successive time-points (baseline to 18 months, *Z* = −5.1, *p* < 0.001; 18–36 months, *Z* = −4.8, *p* < 0.001; 36–54 months, *Z* = −3.4, *p* = 0.001). The MDS-UPDRS III score was significantly greater at baseline compared to 18 months (*Z* = −4.7, *p* < 0.001), 36 months compared to 18 months (*Z* = −4.0, *p* < 0.001) and 54 months compared to baseline (*Z* = −4.8, *p* < 0.001). There was a significant time effect for MoCA [X${}_{\left(3\right)}^{2}$ = 9.9; *p* = 0.02], with the score decreasing from 18 to 54 months (*Z* = −2.5, *p* = 0.013) and from 36 to 54 months (*Z* = −2.9, *p* = 0.004).

**Table 1:** Demographic, cognitive, and clinical characteristics of participants at Baseline, 18, 36, and 54 months.

**PARAMETER**	**Baseline**	**18 months**	**36 months**	**54 months**
Age (years)^*^	65.86 ± 8.27	67.42 ± 8.15	68.86 ± 8.16	70.40 ± 8.18
Sex (Male, Female)	23, 12	23, 12	23, 12	23, 12
Body Mass Index (kgm^−2^)	27.20 ± 3.87	27.41 ± 4.29	27.35 ± 4.49	27.04 ± 4.90
PD duration (years)^*^	0.45 ± 0.33	2.01 ± 0.35	3.45 ± 0.40	4.99 ± 0.52
LEDD^*^	142.8 ± 113.1	337.6 ± 202.5	438.2 ± 227.0	631.5 ± 251.2
Hoehn and Yahr stage^*^	1.71 ± 0.52	2.00 ± 0.48	2.03 ± 0.17	2.14 ± 0.35
UPDRS III^*^	22.46 ± 9.61	28.80 ± 7.15	35.97 ± 10.12	37.11 ± 10.99
ABC	87.72 ± 13.72	85.35 ± 15.66	82.82 ± 19.87	80.87 ± 20.38
MoCA^*^	26.23 ± 2.65	26.89 ± 2.80	26.77 ± 3.25	25.54 ± 3.56
Digit span	6.09 ± 1.20	6.00 ± 1.19	6.17 ± 1.16	5.89 ± 0.95
GDS	2.71 ± 2.47	2.23 ± 2.70	2.63 ± 2.55	3.11 ± 2.25

*PD, Parkinson's Disease; LEDD, Levodopa Equivalent Daily Dose; UPDRS III, Unified Parkinson's Disease Rating Scale Section III; MoCA, Montreal Cognitive Assessment; Digit, Wechsler Forward Digit Span; ABC, Activities Balance Self Confidence Scale; GDS, Geriatric Depression Scale*.

* *significant time effect (p < 0.05)*.

### Accelerometer Metrics

#### Outliers

The metrics of Jerk, RMS, frequency, and ellipsis were analyzed for outliers using the threshold of two standard deviations above or below the mean. Data from these outliers were considered removed from further analysis. Jerk and frequency had the greatest number of outliers across axes, phases, and time-points (1.9%) compared to RMS (1.5%) and ellipsis (1.2%).

### Postural Dynamics

**Figure 1:**
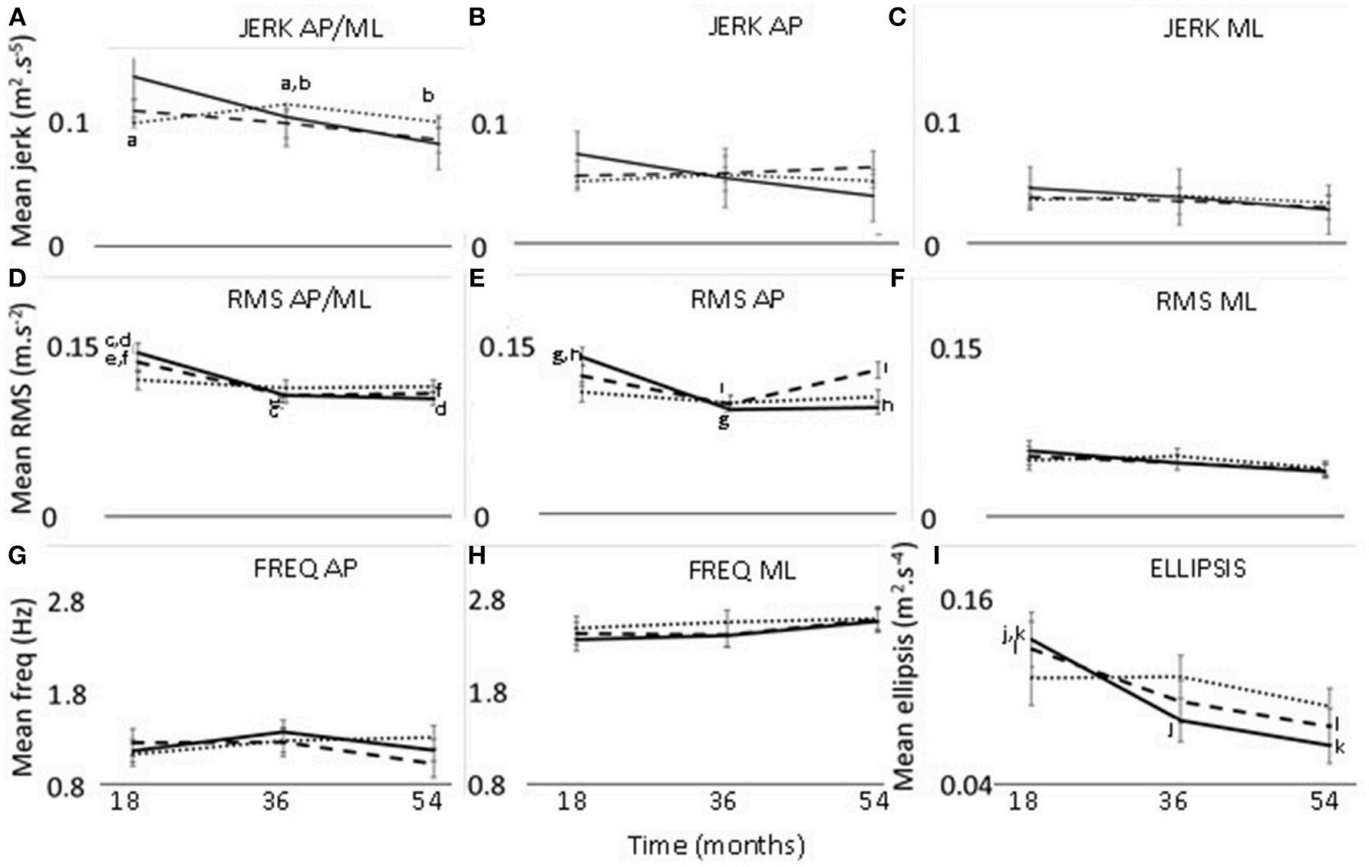
Changes in jerk **(A–C)**, RMS **(D–F)**, frequency **(G,H)**, and ellipsis **(I)** at 18, 36, and 54 months. Thirty seconds postural phase; 60 s postural phase; 120 s postural phase. AP, anteroposterior axis; ML, mediolateral axis; **a-l**—significant difference (*p* < 0.05) between two timepoints.

a) Axis
  The axis had a significant effect on jerk, RMS and frequency (Table 2). Jerk and RMS were greater in the AP direction compared to the ML direction (jerk *p* = 0.001, effect size = 0.87, power = 65.9%; RMS *p* < 0.001, effect size = 2.58, power = 100%) (Figure 1). Frequency was however greater in the ML direction (*p* < 0.001, effect size = 2.60, power = 100%).
b) Time
  There was a significant effect of time for ellipsis, which decreased from 18 to 54 months (*p* = 0.033, effect size = 0.27, power = 45.3%) (Table 2) (Figure 1).

**Table 2:** Mixed linear model results for single and interaction effects of time (18, 36, and 54 months), axis (combined, mediolateral, anteroposterior) and phase (30, 60, 120 s) on gait accelerometry parameters.

	**Jerk**	**RMS**	**Frequency**	**Ellipsis**
Time	NS	NS	NS	*F*_(2, 35)_ = 3.7, *p* = 0.034
Axis	*F*_(2, 22)_ = 107.3, *p* < 0.001	*F*_(2, 57)_ = 286.9, *p* < 0.001	*F*_(1, 34)_ = 223.8, *p* < 0.001	NA
Phase	F_(2, 65)_ = 133.9, *p* < 0.001	NS	NS	NS
Axis × time	NS	*F*_(4, 591)_ = 8.6, *p* < 0.001	NS	NA
Phase × time	NS	*F*_(4, 590)_ = 13.5, *p* < 0.001	NS	*F*_(4, 141)_ = 10.00, *p* < 0.001
Axis × phase × time	*F*_(12, 588)_ = 15.6, *p* < 0.001	*F*_(12, 602)_ = 2.1, *p* = 0.014	NS	NA

*Only findings with p < 0.05 are listed. RMS, Root Mean Square; NS, Not Significant; NA, Not Applicable*.

#### Interaction Effect of Phase and Time

The interaction effect of postural phase and time was significant for RMS and ellipsis (Table 2). Pairwise comparisons showed the RMS and ellipsis parameters for the initial 30 s to be significantly lower at 36 months (RMS *p* = 0.005, effect size = 0.52, power = 63.4%; ellipsis *p* = 0.032, effect size = 0.44, power = 47.7%) and 54 months (RMS *p* = 0.001,effect size = 0.52, power = 77.7%; ellipsis *p* = 0.001, effect size = 0.57, power = 75.6%) compared to 18 months. Additionally, for the initial 60 s there was a decrease from 18 to 54 months for ellipsis (*p* = 0.023, effect size = 0.41, power = 47.8%) (Figure 1).

#### Interaction Effect of Axis and Time

There was a significant interaction effect between axis and time for RMS (Table 2). Pairwise comparisons for the RMS parameter showed that, in the combined direction, 18 months was significantly >36 months (*p* < 0.037, effect size = 0.41, power = 44.1%) and 54 months (*p* = 0.017, effect size = 0.41, power = 49.4%). Along the AP axis, RMS was greater at 18 months compared to 36 months (*p* = 0.022, effect size = 0.43, power = 49.6%).

#### Interaction Effect of Axis, Phase, and Time

The interaction effect of axis, phase, and time was significant for jerk and RMS (Table 2). The 120 s combined jerk parameter was greater at 36 months compared to 18 months (*p* = 0.036 effect size = 0.24, power = 43.4%) and 36 months compared to 54 months (*p* = 0.045 effect size = 0.22, power = 41.1%) (Figure 1A). The RMS parameters along the combined axis decreased significantly from 18 to 36 months for 30 s (*p* = 0.002 effect size = 0.44, power = 70.2%) and 60 s (*p* = 0.022 effect size = 0.35, power = 48.0%) (Figure 1D). These RMS parameters also decreased from 18 to 54 months (30 s *p* < 0.001 effect size = 0.46, power = 83.9%, 60 s *p* = 0.018 effect size = 0.32, power = 50.0%). Additionally, along the AP axis, the 30 s RMS decreased from 18 to 36 months (*p* < 0.001 effect size = 0.56, power = 86.9%) and 54 months (*p* < 0.001 effect size = 0.53, power = 89.6%) (Figure 1E). However, for 60 s RMS increased from 36 to 54 months (*p* = 0.011 effect size = 0.19, power = 51.2%) (Figure 1E).

### Association Between Postural Dynamics and Balance Confidence, Cognitive Function and Depression Measures

Table 3 lists cross-sectional correlations between postural measures of jerk, RMS, and ellipsis in the AP direction and ABC, MOCA, and GDS scores at 36 and 54 months. Few correlations were observed for frequency. No relationship was found at 18 months and only few associations for the first 30 and 60 s of standing. No correlations were present between postural measures and the digit span scores.

a) Jerk
  At 36 months, moderate negative correlations were found for jerk with ABC, MoCA, and a weak positive correlation with GDS. At 54 months, there was a moderate correlation between jerk and ABC and a weak negative correlation with MoCA.
b) RMS
  At 36 months, a moderate negative correlation was observed with ABC, a weak negative correlation between RMS and MoCA and moderate positive correlation with GDS. RMS was moderately negatively correlated with ABC at 54 months and weakly correlated with GDS.
c) Ellipsis
  Moderate negative correlations between ellipsis and ABC at 36 and 54 months were found. There was a positive correlation between ellipsis and GDS at 36 months.

**Table 3:** Spearman's correlation coefficient for mean postural parameters of jerk, RMS, and ellipsis in the AP direction across 120 s with clinical characteristics at 36 and 54 months.

	**Jerk**	**RMS**	**Ellipsis**
	**rho (p)**	**rho (p)**	**rho (p)**
**36 MONTHS**		
ABC	−**0.336 (0.024)**	−**0.462 (0.003)**	−**0.434(0.006)**
MoCA	−**0.392 (0.010)**	−0.291 (0.050)	−0.278 (0.058)
GDS	0.287 (0.047)	**0.380 (0.015)**	**0.433 (0.006)**
**54 MONTHS**		
ABC	−**0.441 (0.004)**	−**0.412 (0.009)**	−**0.455 (0.004)**
MoCA	–0.261(0.065)	–0.113 (0.266)	–0.197 (0.135)
GDS	0.129 (0.230)	0.233 (0.096)	0.215 (0.115)

*ABC, Activities Balance Confidence Scale; MoCA, Montreal Cognitive Assessment; GDS, Geriatric Depression Scale. Moderate Correlations (0.5 < rho>0.3) in bold*.

### Correlation Between Change in Postural Dynamics and Change in Balance Confidence, Cognitive Function, and Depression Measures

**Figure 2:**
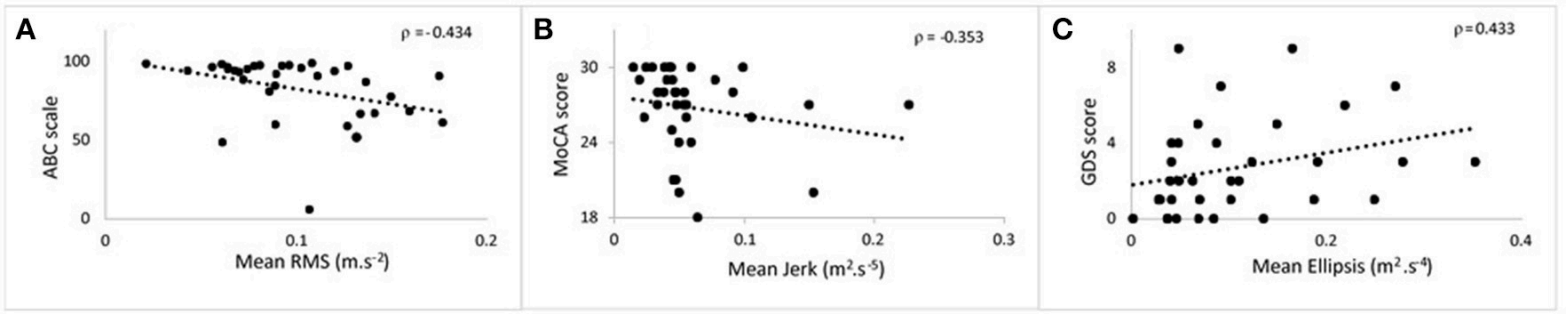
Scatter plots showing the correlation at 36 months between postural parameters and cognitive measures for 120 s phase. **(A)** Mean root mean square against ABC. **(B)** Mean jerk against MoCA. **(C)** Mean ellipsis against GDS. ABC, Activities Balance Self Confidence; MoCA, Montreal Cognitive Assessment; GDS, Geriatric Depression Scale.

**Figure 3:**
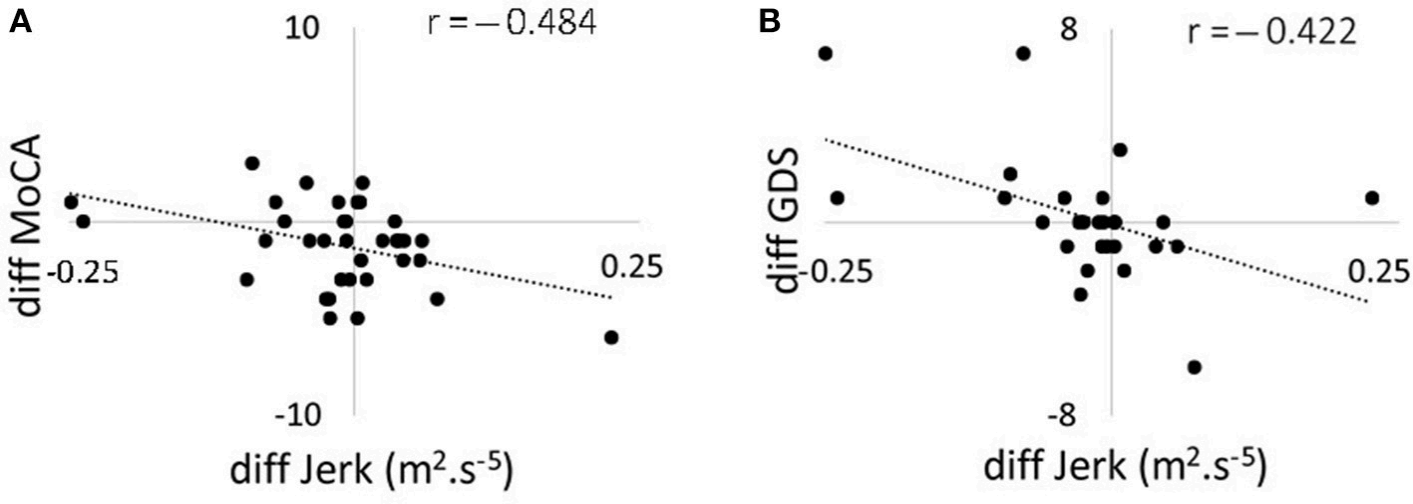
Scatter plots showing the correlation between changes in 120 s jerk from 36 to 54 months and **(A)** changes in MoCA score from 36 to 54 months; **(B)** changes in GDS from 36 to 54 months. MoCA, Montreal Cognitive Assessment; GDS, Geriatric Depression Scale.

Correlations between changes in postural parameters for 120 s in the combined direction and ABC, MoCA, and GDS scores are presented in Table 4 and Figure 2. There were no significant correlations from 18 to 36 months between postural parameters and the ABC, MoCA, and GDS scores. The decline in jerk from 36 to 54 months showed a moderate negative correlation with the change in MoCA (Figure 3A) and a moderate positive correlation with change in GDS scale (Figure 3B).

**Table 4:** Pearson's correlation coefficient for change in mean postural dynamic parameters of 120 s jerk, 30 s RMS, and 30 s ellipsis with change in clinical characteristics.

**Time-points**	**Jerk**	**RMS**	**Ellipsis**
	**r (p)**	**r (p)**	**r (p)**
**18–36 MONTHS**		
ABC	−0.043 (0.406)	0.145 (0.222)	0.092 (0.308)
MoCA	−0.152 (0.192)	−0.052 (0.390)	−0.018 (0.923)
GDS	0.092 (0.299)	−0.140 (0.227)	−0.250 (0.080)
**36–54 MONTHS**		
ABC	−0.204 (0.120)	0.088 (0.318)	−0.064 (0.361)
MoCA	–**0.422 (0.006)**	0.157 (0.200)	0.156 (0.193)
GDS	**0.484 (0.005)**	−0.246 (0.123)	−0.172 (0.201)

*ABC, Activities Balance confidence Scale; MoCA, Montreal Cognitive Assessment; GDS, Geriatric Depression Scale. Moderate Correlations (0.5 < rho>0.3) in bold*.

## Discussion

In this longitudinal study, we aimed to investigate how postural dynamics change during quiet standing in people with PD over 36 months. Additionally, we investigated the relationship between postural dynamics with balance confidence, global cognition and depression score. We hypothesized that postural dynamics of jerk, RMS, and ellipsis would increase, suggesting increased postural instability, with disease progression. However, our novel findings reported mainly a decline in jerk, RMS, and ellipsis with disease progression. Although most change over time for RMS, frequency and ellipsis was observed for the first 30 s of standing, we found that for jerk, significant changes were present only for the entire 120 s duration. We observed significant relationships between postural parameters with balance confidence, global cognition, and depression score suggesting shared neural pathways.

The decrease in RMS is in partial agreement with Mancini et al. (16). Although Mancini et al. (16) reported an increase over 6–9 months in postural parameters in five individuals not receiving dopaminergic medication, eight subjects on dopaminergic medication displayed trends toward decreased RMS (49). Another study, also reported decreased sway (measured with platform mounted potentiometer) in eight patients with moderate PD (Hoehn and Yahr III–IV) on dopaminergic medication, compared to healthy older adults (50). Interpretation of changes in postural dynamics in people with PD involves not only consideration of changes due to progression of pathology but also concomitant age-related neurodegenerative changes. Duarte and Sternad (51) reported older adults show reduced amplitude of postural sway during prolonged standing compared to younger adults (51). However, in a cross-sectional study, Park et al. (52) reported increased postural accelerometry parameters in older adults, apart from frequency and jerk in the ML direction, which decreased (52). It is unclear to what extent longitudinal changes in RMS in people with PD are the consequence of age-related changes rather than due to progression of PD.

The longitudinal decline in RMS and ellipsis from 18 to 54 months was most prominent for the first 30 s of standing. Del Din et al. (17) have reported that this period was most discriminative between people with PD and healthy older adults (17), suggesting that the initial period requires the greatest sensorimotor integration to achieve balance and is most impacted by PD. Theories to explain postural instability in PD include changes in intermittent and continuous control systems (53), impaired proprioception (54), and alteration in awareness of vertical body position relative to the global axes (55). The initial standing period may highlight more the intermittent and continuous control mechanisms needed to adjust the center of mass position to restore stability. Changes in the body position awareness may have a more prominent effect on postural dynamics with increasing standing duration. Dysfunction of sense of body positioning may result in a greater error in return of center of mass to the position optimal for equilibrium and necessitate faster adjustments (greater jerk) to achieve stability. The increase in jerk over 120 s from 18 to 36 months may be the result of faster corrections due to greater error in positioning of center of mass. The subsequent decrease in jerk over 120 s from to 54 months may reflect the complex interaction of disease progression and dopaminergic treatment on postural control mechanisms. There was approximately a 2-fold increase in LEDD from 36 to 54 months compared to 18 to 36 months, which could account for the decrease in 120 s jerk.

There was a significant effect of direction, with the AP direction showing greater change than ML, with a decrease in RMS value from 18 to 54 months. This finding is supported by a study that reported decreased AP sway during standing in people with PD compared to controls (50). A recent study analyzing postural data from a similar cohort of individuals with PD observed increased regularity of postural dynamics from 18 to 54 months along the AP axis suggesting possible modification of motor control along this axis (56). The reduction in AP postural dynamics may result from greater instability in the AP direction, associated with decreased knee flexion and greater difficulty initiating ankle dorsiflexion to maintain balance (57). Specifically, greater instability has been observed in backward sway when people had normal foot width (57). Axial stiffness increases with PD progression and patients frequently develop a stooped posture (camptocormia) which may also affect AP trunk dynamics (58). However, camptocormia usually presents after 7–8 years of diagnosis, so this is not likely to be a major consideration in our study. The decreased RMS in the AP direction may represent a compensation strategy to maintain stability by keeping movements of the trunk within a smaller, safer range along the AP axis.

We observed negative correlations between postural dynamics with the ABC scale. Greater confidence in standing and a lower fear of falling were associated with lower postural parameters. Reduction in postural dynamics may result from increased lower limb rigidity. Carpenter et al. (59) have reported co-contraction of leg muscles with consequential increased ankle stiffness in people with PD compared to age-matched controls (59). Older adults have also been observed to have increased muscle co-activation compared to young adults with the subgroup of fallers having greater postural sway. Increased co-activation of lower limb agonists and antagonists will result in a more rigid structure, although less reactive to external perturbations. We did not find significant change with disease progression for postural parameters along the ML axis. One study reported increased RMS in the ML direction in people with PD compared to healthy older adults, therefore the RMS value might be expected to increase as the disease progressed (60). However, the observation that there is no longitudinal change may be the result of the interaction of opposing age-related and PD effects on the control of postural dynamics.

We found moderate negative correlations between MoCA and postural dynamics at the 36 months time point with lower cognition associated with increased jerk. Kelly et al. (61) have reported similar findings between lower global cognition and increased postural instability (61). Correlation between postural measures and cognitive tests have also been reported by Nocera et al. (62). Dysfunctions in dopamine networks may to some extent account for this association as impairment in executive function and attention is mediated partially by dopaminergic frontostriatal networks (63). No relationship between postural and cognitive measures was observed at 54 months, which may be due to progression of the pathology, emergence of additional clinical features and effect of medication. Levodopa has been suggested to improve some balance measures but worsen others (64) and in the advanced stages of PD increases postural sway (65). Although both jerk and MoCA decreased on average from 36 to 54 months, we found a moderate negative correlation in the difference between the two time-points for these parameters. The mean change in the MoCA score was 1.23 and reduced the MoCA score at 54 months to 25.54, which is considered clinically to indicate possible cognitive impairment. Our finding suggests there individuals with an increase in postural parameters decrease their cognitive function. This is surprising as mild cognitive impairment is associated with postural instability (66) and increased rates of cognitive decline have been reported in individuals with postural instability and gait disturbance phenotype (67). Possible different effects of disease progression and aging on postural dynamics and cognitive function may explain the results. Further investigation is however needed.

There were moderate positive correlations between GDS score and postural dynamics. The changes in GDS were however small, and even at 54 months the GDS at 3.11 was below the threshold of 5 which has been reported to indicate mild depression. Previous studies have reported a relationship between depression and gait parameters, which was stronger on dopaminergic medication (68, 69). A review of depression in PD reported contradictory findings regarding postural correlates of depression in PD (29). The association between GDS score and postural instability may be related to the physical constraint on activity imposed by postural instability because of the increased falls risk. However, as depression frequently precedes the motor symptoms, the association is more likely to be due to changes in shared neural circuitry (70).

### Limitations

The main limiting factor is that we tested patients in the ON medication state. Postural dynamics will differ in the OFF state compared to the ON state, with motor impairment reported to be greater in the OFF state.

Our standing balance test involved participants self-selecting their foot position. This may from 18 to 54 months consequential decrease in postural dynamics. By contrast, many postural studies adopt a standardized foot position, which restricts patients changing their base of support. However, the purpose of our postural analysis was to examine individual postural dynamics by allowing participants to wear their own comfortable footwear and place their feet in a position they considered would provide them with maximum stability.

## Conclusion

Postural dynamics decrease over a period of 36 months in people with PD. This may be due to people reducing their postural sway in order to restrict their center of pressure excursions to a smaller “safe” area, as postural instability increases with disease progression. Underlying mechanisms may include co-contraction of agonist and antagonist muscles resulting in increased rigidity. Limiting postural movements may however result in the individual becoming less able to respond to external perturbations and therefore becoming more prone to falls. Postural dynamics are associated at 36 months after diagnosis, with global cognition and depression, emphasizing the interaction between motor and non-motor features, which may reflect shared neural correlates as the locus coeruleus. This study demonstrates the multisystem nature of PD and the need to examine different features as part of a whole unified system.

## Ethics Statement

This study was carried out in accordance with the recommendations of the Newcastle and North Tyneside research ethics committee with written informed consent from all subjects. All subjects gave written informed consent in accordance with the Declaration of Helsinki. The protocol was approved by the Newcastle and North Tyneside research ethics committee.

## Author Contributions

AP carried out data analysis, interpreted the data, and wrote the manuscript. PS and LK contributed to data analysis and critically reviewed the manuscript. RM was involved with data collection, reviewed, and commented on manuscript draft. AY was involved with data collection and critically reviewed the manuscript. SD developed code, analyzed data, and critically reviewed the manuscript. LR conceived and designed the study, interpreted the data and critically revised the manuscript.

### Conflict of Interest Statement

The authors declare that the research was conducted in the absence of any commercial or financial relationships that could be construed as a potential conflict of interest.
